# Potential reduction of lung dose via VMAT with jaw tracking in the treatment of single‐isocenter/two‐lesion lung SBRT

**DOI:** 10.1002/acm2.12580

**Published:** 2019-04-05

**Authors:** Damodar Pokhrel, Lana Sanford, Matthew Halfman, Janelle Molloy

**Affiliations:** ^1^ Department of Radiation Medicine Medical Physics Graduate Program University of Kentucky Lexington KY USA

**Keywords:** lung SBRT, VMAT, jaw tracking, single‐isocenter/two‐lesion

## Abstract

**Purpose/objectives:**

Due to higher radiosensitivity, non‐target normal tissue dose is a major concern in stereotactic body radiation therapy (SBRT) treatment. The aim of this report was to estimate the dosimetric impact, specifically the reduction of normal lung dose in the treatment of single‐isocenter/two‐lesion lung SBRT via volumetric modulated arc therapy with jaw tracking (JT‐VMAT).

**Materials/methods:**

Twelve patients with two peripherally located early‐stage non‐small‐cell‐lung cancer (NSCLC) lung lesions underwent single‐isocenter highly conformal non‐coplanar JT‐VMAT SBRT treatment in our institution. The mean isocenter to tumors distance was 5.6 ± 1.9 (range 4.3–9.5) cm. The mean combined planning target volume (PTV) was 38.7 ± 22.7 (range 5.0–80.9) cc. A single isocenter was placed between the two lesions. Doses were 54 and 50 Gy in three and five fractions, respectively. Plans were optimized in Eclipse with AcurosXB algorithm utilizing jaw tracking options for the Truebeam with a 6 MV‐FFF beam and standard 120 leaf millennium multi‐leaf collimators. For comparison, the JT‐VMAT plans were retrospectively re‐computed utilizing identical beam geometry, objectives, and planning parameters, but without jaw tracking (no JT‐VMAT). Both plans were normalized to receive the same target coverage. The conformity and heterogeneity indices, intermediate‐dose spillage [D_2cm_, R50, Gradient Index (GI), Gradient Distance (GD)], organs at risks (OAR) doses including normal lung as well as modulation factor (MF) were compared for both plans.

**Results:**

For similar target coverage, GI, R50, GD, as well as the normal lung V5, V10, V20, mean lung dose (MLD), and maximum dose received by 1000 cc of lungs were statistically significant. Normal lung doses were reduced by 8%–11% with JT‐VMAT. Normal lung dose increased as a function of tumor distance from isocenter. For the other OAR, up to 1%–16% reduction of non‐target doses were observed with JT‐VMAT. The MF and beam‐on time were similar for both plans, however, MF increased as a function of tumors distance, consequently, delivering higher dose to normal lungs.

**Conclusion:**

Utilizing jaw tracking options during optimization for single‐isocenter/two‐lesion lung SBRT VMAT plans reduced doses to the normal lung and other OAR, reduced intermediate‐dose spillage and provided superior/similar target coverage. Application of jaw tracking did not affect delivery efficiency and provided excellent plan quality with similar MF and beam‐on time. Jaw tracking is recommended for future clinical SBRT plan optimization.

## 1 INTRODUCTION

Recent advances in stereotactic body radiotherapy (SBRT) technology have greatly improved the ability to deliver conformal therapeutic tumor dose with a biological effective dose (BED) of greater than 100 Gy while minimizing the dose to the adjacent organs at risk (OAR).^1^, ^2^, ^3^ Several studies have shown that safely delivering a higher BED to the lung lesions improved therapeutic ratio and local control rates.^4^, ^5^, ^6^, ^7^, ^8^, ^9^, ^10^ In addition, utilizing volumetric modulated arc therapy (VMAT) planning with a flattening filter free (FFF) beam in lung SBRT treatment reduced the total number of monitor units (MUs)^11^, ^12^ and the treatment time compared to intensity modulated radiotherapy, Tomotherapy, or CyberKnife.^13^, ^14^, ^15^, ^16^ Reduction in MUs provides faster treatment delivery that can improve patient comfort, decrease potential setup/motion related errors and promote efficient clinical workflow. Owing to those advantages, VMAT SBRT planning using single isocenter for multiple targets has been gaining popularity in clinics for treating multiple intracranial tumors^17^, ^18^ as well as extracranial oligometastases lesions.^19^, ^20^, ^21^, ^22^, ^23^

Conversely, VMAT averages the dose delivery over more angles and produces slightly higher non‐target low dose distribution compared to intensity modulated radiation therapy (IMRT). Generally, the treatment fields are designed with the jaw apparatus and tertiary multi‐leaf collimators (MLCs) shaping the target volume. The jaw apparatus is fixed on the maximum field size of MLCs during treatment delivery, and thus leakage and transmission of radiation through the MLCs is present in the optimized IMRT/VMAT plan. This effect is noticeable while utilizing single‐isocenter/multitarget VMAT plan. When the isocenter to tumor distance is large (on the order of 4–10 cm), the MLCs have to travel a longer distance to provide the target coverage to each lesion, potentially delivering higher non‐trivial low‐dose spillage to the non‐target tissues such as normal lungs. Due to the higher radiosensitivity, non‐target normal tissue dose is one of the major concerns for SBRT treatments.^24^, ^25^, ^26^, ^27^ However, if the jaws move to track MLC positions (called jaw tracking, JT technique on Truebeam), the radiation transmitted, and leakage dose to the normal tissues can be reduced.

Although the advantages of JT‐IMRT/VMAT plans with flattened beams have been studied previously,^28^, ^29^, ^30^, ^31^, ^32^ the dosimetric impact of JT technique with FFF beam in the treatment of lung SBRT patients, along with the treatment delivery complexity due to the use of JT with MLC motion has not yet been reported. The goals of this project were to quantify the dosimetric differences of JT technique for FFF beam in the SBRT treatment of multifocal lung lesions and to investigate the JT delivery complexity with MLC movements. In this report we retrospectively evaluated 12 single‐isocenter/two‐lesion early stage NSCLC patient's plans who underwent SBRT treatment in our clinic using JT‐VMAT. For those patients, the non‐target low dose was minimized by using jaw tracking options for the Truebeam Linac with a 6 MV‐FFF beam (in Eclipse treatment planning system (TPS), Varian Medical System, Palo Alto, CA) during SBRT VMAT plan optimization. For comparison, the clinical JT‐VMAT plans were re‐computed without jaw tracking (no‐JT‐VMAT) options. The original clinical JT‐VMAT and no‐JT‐VMAT plans were compared via lung SBRT protocol compliance criteria, target conformity, gradient indices, dose to lungs, and other OAR per RTOG guidelines.

## 2 MATERIALS AND METHODS

### 2.A Computed tomography (CT) simulation and contouring

A total of 12 sequential patients who underwent single‐isocenter/two‐lesion lung SBRT treatment in our clinic were included in this retrospective study, all of whom had two peripherally located Stage I NSCLC lesions. The patients were immobilized using Body Pro‐Lok^™^ platform (CIVCO system, Orange City, IA) in the supine position, arms above their head with abdominal compression. All patients received four‐dimensional (4D)‐CT scan using Varian's Real Time Position Management Respiratory Gating System (version 1.7) in addition to conventional three‐dimensional (3D) CT scan on a GE Lightspeed 16 slice CT scanner (General Electric Medical Systems, Waukesha, WI). CT images were acquired with 512 × 512 pixels at 2.5 mm slice thickness in the axial cine mode. All 10 phases of 4D CT slices and respiratory motion signal were transferred to an Advantage 4D Workstation (General Electric Medical Systems, San Francisco, CA), where the maximum intensity projection (MIP) images were generated after phase binning of the 4D CT images. In addition to the MIP images, a physicist confirmed the motion of both tumors was less than 1 cm. The regular 3D CT scan and the MIP images were imported into the Eclipse TPS (version 13.0, Varian Medical Systems, Palo Alto, CA) and co‐registered for target contouring. Gross tumor volumes (GTV) and internal tumor volumes (ITV) were delineated on the 3D CT images with reference to the MIP images. Planning target volumes (PTV) were generated by adding non‐uniform 5–10 mm margins to the ITV to accommodate the patient setup uncertainties based on tumor size, location, and synchronous tumor motion. The critical structures, such as bilateral lungs excluding the ITV (normal lung), spinal cord, ribs, heart, great vessels, esophagus, and skin were delineated on the 3D CT images.

The tumor characteristics for the single‐isocenter/two‐lesion lung SBRT patients are summarized in Table 1, including isocenter to tumors distance, normal lung volume, and tumor location. The combined PTV was defined as PTV1 plus PTV2. Both lesions were treated synchronously with a total dose of 54 Gy or 50 Gy in three and five fractions, respectively. Normal lung volume ranged from 1893 to 6543 cc, mean 3881 cc. The average value of isocenter to tumors distance was 5.6 cm (range 3.4 to 9.5 cm).

**Table 1:** Characteristics of single‐isocenter/two‐lesion lung stereotactic body radiation therapy (SBRT) patients treated with volumetric modulated arc therapy with jaw tracking (JT‐VMAT) plan included in this study

Parameters	Mean ± SD (range or no. of patients)
Lesion 1, PTV1 (cc)	21.5 ± 20.7 (5.0–80.9)
Lesion 2, PTV2 (cc)	17.2 ± 10.7 (7.7–43.6)
Combined PTV (cc)	38.7 ± 22.7 (15.9–91.8)
Prescription dose (each lesion)	54 Gy in three fractions (six patients)
	50 Gy in five fractions (six patients)
Normal lung volume (cc)	3881 ± 1161 (1893–6543)
Isocenter to tumors distance (cm)	5.6 ± 1.9 (3.4–9.5)
Tumor location	Left lung lesions (four patients)
	Right lung lesions (two patients)
	Bilateral lungs lesions (six patients)

### 2.B Treatment planning

#### 2.B.1 Clinical single‐isocenter JT‐VMAT plan

Highly conformal, clinically optimal VMAT treatment plans were generated using 3–5 non‐coplanar partial arcs (5–10^°^, couch kicks were used for arcs) for the Truebeam linear accelerator (Varian, Palo Alto, CA) with millennium MLC and a 6 MV‐FFF (1400MU/min) beam. A single isocenter was placed approximately between the two lesions in each patient. For each arc, collimator angles were chosen such that the opening of the MLC between tumors was minimized. Additionally, the jaw tracking (JT) option was chosen during plan optimization to further minimize the non‐target dose. A dose of 54 or 50 Gy in three and five fractions was prescribed to the PTV of which D95% received at least 100% of the prescription. All hot spots were within each ITV (i.e., the center of each ITV was 20% hotter). All clinical treatment plans were calculated using the Eclipse TPS with Acuros XB (version 13.6.0, Varian Medical Systems, Palo Alto, CA) algorithm on the 3D CT images with heterogeneity corrections using a 2.0 × 2.0 × 2.0 mm^3^ dose calculation grid‐size. Dose to medium was reported. All clinical plans were inversely optimized using variation of gantry rotation speed, dose rate, and MLC positions. In addition to optimization ring structures, the generalized normal tissue objective (NTO) parameters were used to control the gradients for each target. Planning objectives were per RTOG 0915 guidelines. These patients were treated every other day per lung SBRT protocol.

#### 2.B.2 Quality assurance and treatment delivery

For each plan, a verification plan was generated in the Eclipse TPS using an Octavius phantom (PTW, Freiburg, Germany). Doses re‐calculated on the phantom's 2D ionization chamber array were exported and compared to a measured dose distribution. Using the *γ*‐evaluation method of VeriSoft (Version 6.3, PTW) the two distributions were compared using the standard clinical gamma passing rate criteria of 3%/3 mm maximum dose difference and distance‐to‐agreement with 10% threshold as well as maximum point dose. The Octavius QA pass rates for the single‐isocenter/two‐lesion lung SBRT plan were 98.8 ± 2.5%, on average, for 3%/3 mm clinical gamma pass rate criteria and the maximum point dose measurement was 1.0 ± 0.7%, on average, suggesting that lung SBRT plans using JT can be accurately delivered. The beam‐on time was estimated by using dose rates of 1400 MU/min for these plans. The dose‐rate was confirmed by reviewing each VMAT arc for all patients under the MLC properties in Eclipse. Additionally, maximum dose rate of 1400 MU/min was visually observed during VMAT QA delivery at Truebeam for all single‐isocenter/two‐lesion lung SBRT plans.

Before delivering each JT‐VMAT SBRT treatment, a daily quality assurance check on kilovoltage to megavoltage imaging isocenter coincidence was performed, including IsoCalc measurement for precise and accurate target localization. Our IsoCalc localization accuracy for Truebeam was <0.5 mm. All the quality assurance procedures were in compliance for SBRT treatment delivery. The patients received daily cone beam CT per image‐guidance procedures established in our clinic.

#### 2.B.3 No JT‐VMAT plan

The JT‐VMAT SBRT treatment plans for all patients were retrospectively computed with a no JT‐VMAT approach. All the planning objectives used in the no JT‐VMAT were identical to the JT‐VMAT plan including the NTO parameters and ring structures. The no JT‐VMAT SBRT plan received the same target coverage as the JT‐VMAT plan. Dosimetric parameters for the target coverage and the dose to adjacent OAR including normal lung doses were evaluated.

### 2.C Plan evaluation

The dose volume histograms (DVHs) and isodose curves of JT‐VMAT vs no JT‐VMAT plans were compared. The Conformity index (CI), heterogeneity index (HI), gradient index (GI), gradient distance (GD), and D_2cm_ were calculated per RTOG 0915 recommendations. The dose to the normal lung was evaluated using V5, V10, V20, mean lung dose (MLD), and maximum dose to 1000 cc of lungs. Furthermore, dosimetric disparities were evaluated for spinal cord, heart, bronchial tree, esophagus, trachea, ribs, and skin following RTOG guidelines. The mean and standard deviation values for each of the dose metrics were compared using paired *t* tests for JT‐VMAT vs no JT‐VMAT using *P* < 0.05.

To estimate the normal lung dose as a function of target distance from the single isocenter, the isocenter to tumor distance was calculated as the maximum 3D‐linear distance from the isocenter to the geometric center of each tumor. This distance was calculated in the Eclipse TPS using the x‐, y‐, and z‐ primary coordinates of the tumor centers. Moreover, the modulation factor (MF) as a function of isocenter to tumor distance was evaluated by using total number of monitor units (MUs) delivered for the both JT‐VMAT and no‐JT VMAT SBRT plans. The MF is defined as the total number of MUs divided by the prescription dose in cGy.

## 3 RESULTS

### 3.A Targets coverage

**Figure 1:**
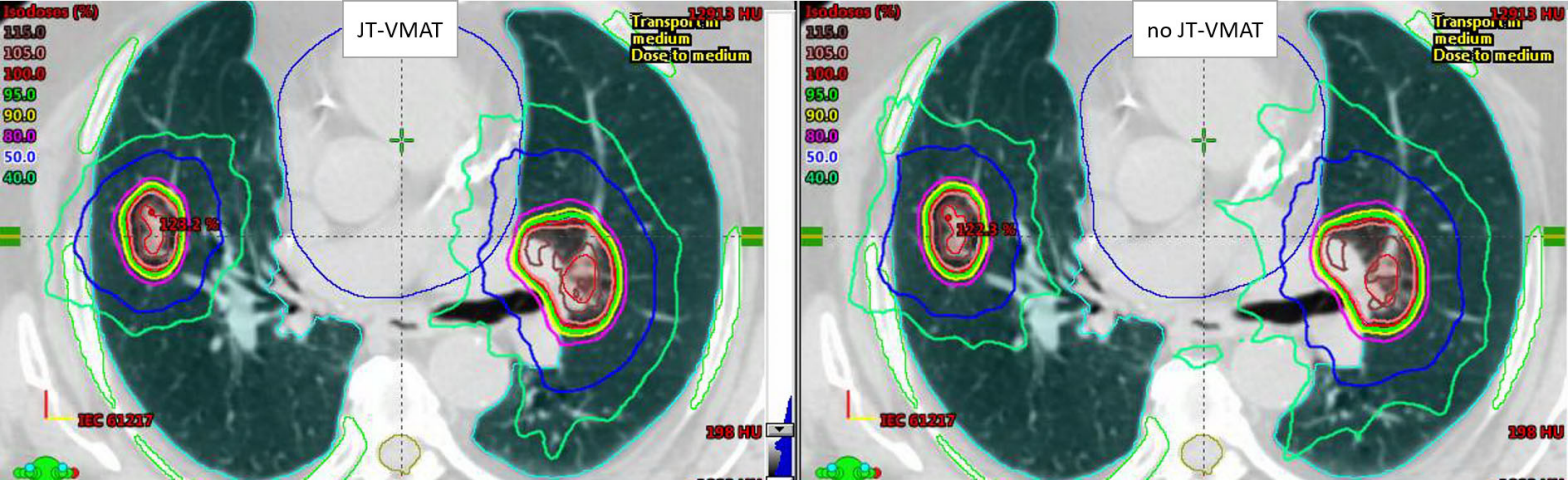
Comparison of dose distributions for a patient with two lung lesions treated with single‐isocenter volumetric modulated arc therapy with jaw tracking (JT‐VMAT) stereotactic body radiation therapy (SBRT) plan (left panel). The single‐isocenter location is shown by the cross‐hair. This patient received a synchronous SBRT treatment to a total dose of 50 Gy to each lesion in five fractions. Tumors were located in bilateral lungs. Isocenter to tumors distance was about 8 cm. Lesion 1, planning target volume (PTV)1 (left lung) = 80 cc and lesion 2, PTV2 (right lung) = 11 cc. For the similar target coverage, conformity, and heterogeneity, the intermediate‐dose spillage (see 40% isodose lines corresponding to 20 Gy dose on both plans) was tighter (more clinically shaped) with volumetric modulated arc therapy with jaw tracking (JT‐VMAT; left panel) compared to no JT‐VMAT (right panel).

**Figure 2:**
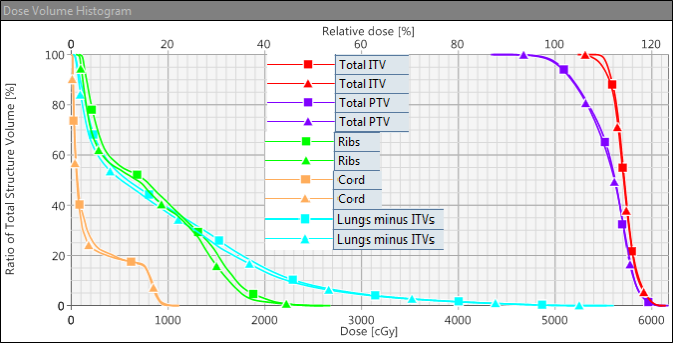
Dose volume histograms (DVHs) comparison between volumetric modulated arc therapy with jaw tracking (JT‐VMAT) and no JT‐VMAT plans for an example case shown in Fig. 1. As shown above, this patient received a single‐isocenter/two‐lesion JT‐VMAT plan. Square markers show DVH for no JT‐VMAT, triangle markers show DVH calculated with JT‐VMAT and demonstrate that combined planning target volume (PTV; purple color) and combined internal tumor volumes (ITV; red color) had an identical target coverage. Ribs (green color) and spinal cord (light orange color) DVHs are also shown as well as lungs minus ITVs (light blue color). The clinical JT‐VMAT significantly reduced low‐dose spillage to the normal lungs.

Both plans were normalized to receive the same target coverage (i.e., PTVD95 = 100%). Although jaw tracking was applied for JT‐VMAT compared to no JT‐VMAT plan, the dose distribution in the target volumes remained comparable with no significant differences in conformity and uniformity indices, as shown in Table 2. An example isodose distribution and DVHs are shown in Figs. 1 and 2, respectively. Although both plans were acceptable per the RTOG standard, the JT‐VMAT plan had advantages of providing tighter intermediate‐dose spillage (see R50, GI, and GD, significant *P*‐values in Table 2) compared to no JT‐VMAT plan.

**Table 2:** Plan quality evaluation for single‐isocenter/two‐lesion lung stereotactic body radiation therapy (SBRT) volumetric modulated arc therapy with jaw tracking (JT‐VMAT; clinical) and no JT‐VMAT (re‐planned) plans for all 12 patients

Target volume	Parameters	JT‐VMAT	No JT‐VMAT	*P*‐value
Combined PTV	CI	1.04 ± 0.02	1.05 ± 0.03	*n.s*.
	HI	1.17 ± 0.02	1.17 ± 0.03	*n.s*.
	R50 (%)	5.30 ± 0.88	5.47 ± 0.92	***P = 0.001***
	D_2cm_ (%)	55.18 ± 6.30	55.43 ± 5.94	*n.s*.
	GI	5.12 ± 0.82	5.26 ± 0.87	***P = 0.001***
	GD (cm)	1.46 ± 0.16	1.49 ± 0.18	***P = 0.0002***

Combined planning target volume (PTV) = PTV1 plus PTV2. CI = conformity index, total volume covered by the 100% isodose line divided by the volume of the combined PTV. HI, heterogeneity index = D10%/D95%, where D10% is the dose to the hottest 10% of the combined PTV and D95% is the dose to the 95% of the combined PTV coverage. R50 (%) = ratio of 50% prescription isodose volume to the combined PTV. D2 cm (%) = maximum dose (in % of dose prescribed) 2 cm away from PTV in any direction. GI = R50%/R100%, R50% is the ratio of 50% prescription isodose volume to the combined PTV and R100% is the ratio of 100% prescription isodose volume to the combined PTV. GD (cm) = is the average distance from 100% prescription dose to 50% of the prescription dose. Statistically significant *P*‐values are in bold, n.s.  = not significant.

### 3.B Dose to lungs

The absolute differences between single‐isocenter JT‐VMAT and no JT‐VMAT SBRT plans for normal lung V20, V10, V5, MLD, and the maximum dose received by 1000 cc of lungs are listed in Table 3. All patients had V20 < 10%–15% for JT‐VMAT treatment plans per protocol. The absolute differences of V20, V10, and V5 were up to 2%, 3%, and 4% higher, respectively with no JT‐VMAT plans. Doses to all lung parameters increase uniformly with no JT‐VMAT plan compared to JT‐VMAT plan, giving statistically significant differences (*P* = 0.002, 0.003, 0.001, 0.001, and 0.001, respectively). Statistically significant *P*‐values are in bold (see Table 3).

**Table 3:** Normal lung dose statistics between single‐isocenter/two‐lesion volumetric modulated arc therapy with jaw tracking (JT‐VMAT) and no JT‐VMAT plans for all 12 lung stereotactic body radiation therapy (SBRT) patients. Mean ± standard deviation (range) and *P*‐values were presented

Plan type	V20 (%)	V10 (%)	V5 (%)	MLD (Gy)	Maximum dose to 1000 cc of lungs (Gy)
JT‐VMAT	6.6 ± 3.5 (2.9 to 13.5)	18.5 ± 8.6 (8.2 to 36.8)	31.3 ± 11.4 (15.4 to 50.4)	5.6 ± 3.5 (3.0 to 9.2)	6.2 ± 3.1 (2.3 to 11.2)
No JT‐VMAT	7.3 ± 3.9 (3.0 to 15.4)	20.3 ± 9.5 (8.4 to 39.1)	33.6 ± 12.4 (16.0 to 53.6)	6.1 ± 2.1 (3.2 to 9.9)	6.9 ± 3.5 (2.6 to 12.9)
No JT‐VMAT minus JT‐VMAT	0.7 ± 0.6 (0.2 to 1.9)	1.9 ± 1.7 (0.2 to 6.5)	2.3 ± 1.9 (0.6 to 6.5)	0.5 ± 0.4 (0.2 to 1.6)	0.7 ± 0.6 (0.3 to 2.1)
*P*‐value	***P = 0.002***	***P = 0.003***	***P = 0.001***	***P = 0.001***	***P = 0.001***

Statistically significant *P*‐values are in bold.

**Figure 3:**
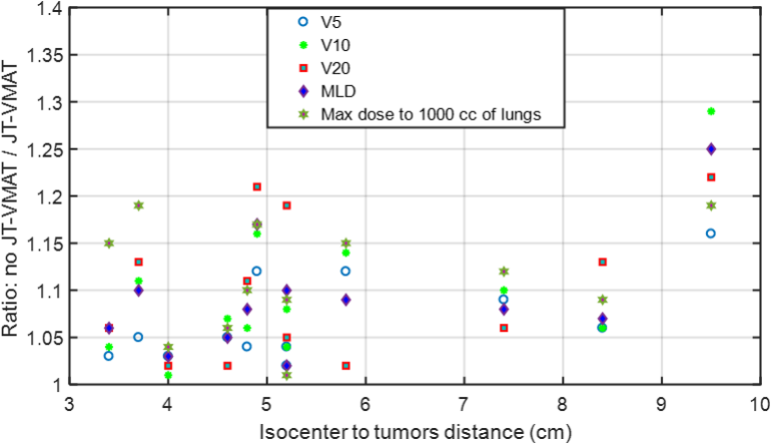
Scatter plot: Ratios of normal lungs V5, V10, V20, MLD, and maximum dose to 1000 cc of lungs calculated by volumetric modulated arc therapy with no jaw tracking (no JT‐VMAT) and JT‐VMAT plans as a function of isocenter to tumor distance. For the identical plan parameters and objectives, the no JT‐VMAT plans gave higher V5, V10, V20, MLD and maximum dose to 1000 cc of lungs by 6%, 8%, 8%, 8%, and 11%, on average, respectively, compared to JT‐VMAT plans.

The variation of ratios between no JT‐VMAT and JT‐VMAT as a function of isocenter to tumor distance for V5, V10, V20, MLD, and maximum dose to 1000 cc of lungs including absolute differences is shown in Fig. 3. For identical planning objectives and optimization parameters, V5, V10, V20, MLD, and maximum dose to 1000 cc of lungs were uniformly higher by 6% (range, 2%–16%), 8% (range, 2‐29%), 8% (range, 2%–22%), 8% (range, 3%–25%), and 11% (range, 2%–19%), on average, respectively, compared to clinical JT‐VMAT plan. In terms of absolute differences, V20, V10, V5, and MLD were higher by up to 1.9%, 6.5%, 6.5%, and 1.6 Gy (in some cases) with no JT‐VMAT compared to JT‐VMAT, respectively. This could be explained by the fact that MLC transmission contributed low‐dose spillage in the normal lung due to MLC traveling longer distances (as a function of isocenter to tumor distance) to provide the same target coverage.

### 3.C Dose to other OAR

A comparison of other OAR dosimetric parameters for single‐isocenter/two‐lesion JT‐VMAT and no JT‐VMAT plans for all 12 lung SBRT patients is presented in Table 4. Critical organs such as spinal cord (D_max_, and D_0.35cc_), heart (D_max_ and D_15cc_), esophagus (D_max_ and D_5cc_), bronchial tree (D_max_), trachea (D_max_ and D_4cc_), ribs (D_max_ and D_1cc_), and skin (D_max_ and D_10cc_) were evaluated per SBRT protocol guidelines.

**Table 4:** Average values and ranges of absolute dose differences between volumetric modulated arc therapy with no jaw tracking (no JT‐VMAT) vs JT‐VMAT plans for the major dose distribution parameters of the other OAR for all 12 lung stereotactic body radiation therapy (SBRT) patients

OARs	Parameters	Mean ± SD (Gy)	Range (Gy)	Ratio^a^	*p*‐value
Spinal cord	D_max_	0.2 ± 0.9	−1.7 to 3.4	1.02 ± 0.12	*n.s*.
	D_0.35cc_	0.2 ± 0.7	−0.6 to 2.1	1.03 ± 0.10	*n.s*.
Heart	D_max_	0.2 ± 1.3	−1.7 to 3.0	1.02 ± 0.08	*n.s*.
Esophagus	D_15cc_	0.5 ± 0.6	−0.6 to 1.4	1.04 ± 0.06	***P = 0.01***
	D_max_	‐0.3 ± 2.0	−4.3 to 3.4	1.01 ± 0.14	*n.s*.
Bronchial tree	D_5cc_	0.2 ± 0.6	−0.5 to 1.4	1.02 ± 0.07	*n.s*.
	D_max_	0.7 ± 1.6	−1.7 to 3.4	1.06 ± 0.13	*n.s*.
Trachea	D_max_	0.4 ± 1.1	−1.1 to 3.0	1.06 ± 0.12	*n.s*.
	D_4cc_	0.6 ± 0.8	0.0 to 2.4	1.16 ± 0.12	***P = 0.02***
Ribs	D_max_	‐0.2 ± 1.7	−4.1 to 2.0	0.99 ± 0.05	*n.s*.
	D_1cc_	0.1 ± 0.9	−2.4 to 1.5	1.00 ± 0.03	*n.s*.
Skin	D_max_	0.2 ± 1.6	−3.3 to 2.4	1.01 ± 0.09	*n.s*.
	D_10cc_	0.3 ± 0.4	−0.6 to 0.9	1.03 ± 0.03	***P = 0.01***

Absolute dose differences = no JT‐VMAT ‐ JT‐VMAT. The negative sign indicates that the results of the JT‐VMAT plans were larger than those of single‐isocenter plans. Mean ± standard deviation, range, and *P*‐values were presented. Statistically significant *P*‐values are in bold. OAR: organs at risks.

a Ratio = no JT‐VMAT/JT‐VMAT and n.s. = not significant.

It was observed that the volumetric dose difference to heart, trachea, and skin were statistically significant (*P*‐values, 0.01, 0.02, and 0.01, respectively) between the two plans. Overall, the doses with no JT‐VMAT SBRT were higher by 1%–16% for the most of the critical organs, suggesting that the average values of absolute dose differences could be higher with no JT‐VMAT plan of the order of 1–2 Gy compared to clinical JT‐VMAT plan.

### 3.D Modulation factor and beam‐on time

**Figure 4:**
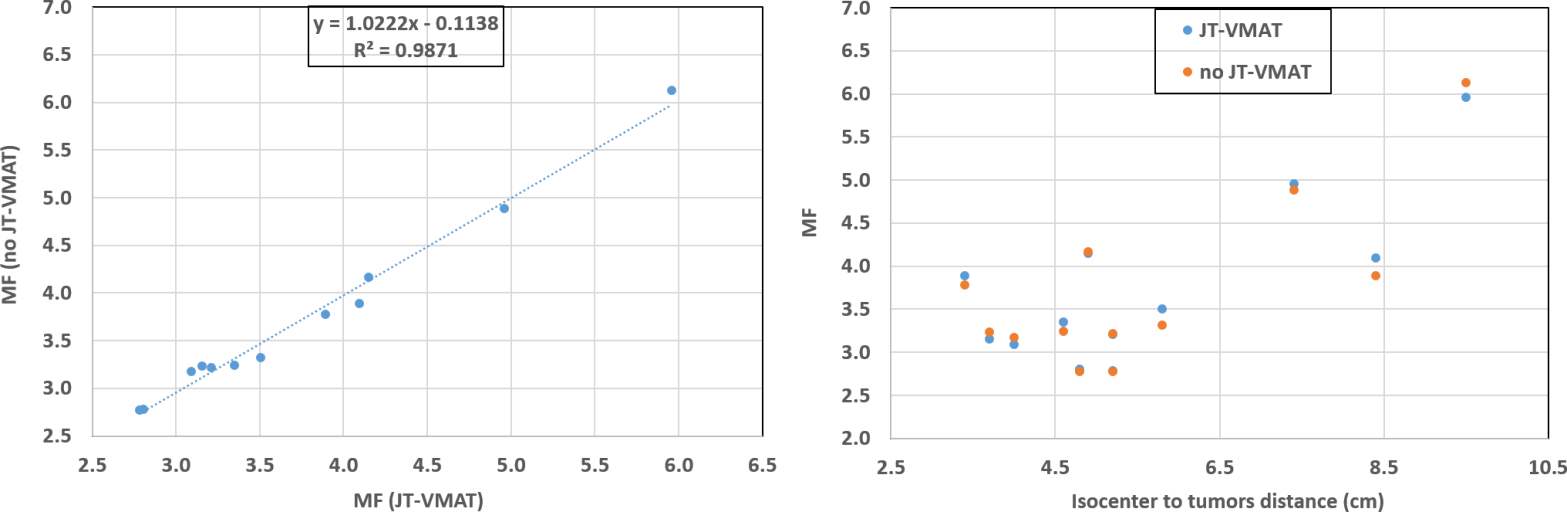
Scatter plots: volumetric modulated arc therapy with no jaw tracking (no JT‐VMAT) modulation factor (MF) as a function of JT‐VMAT (left panel) and MFs as a function of isocenter to tumor distance (right panel) for all 12 single‐isocenter/two‐lesion lung stereotactic body radiation therapy patients. The JT‐VMAT did not change the total number of MUs or delivery efficiency compared to no JT‐VMAT (see left panel).

The MF for no JT‐VMAT vs JT‐VMAT and the MF as a function of the isocenter to tumor distance is shown in Fig. 4. For the given lung SBRT plan, the total number of MUs did not change significantly while using JT options for plan optimization, suggesting that the both plans gave similar MF. The average values of the MF for no JT‐VMAT vs JT‐VMAT were 3.72 ± 0.97 vs 3.75 ± 0.94, respectively. The average beam on time for JT‐VMAT plan was 3.8 ± 1.7 min similar to that of no JT‐VMAT plan (3.7 ± 1.1 min) thus not affecting the beam‐on time, significantly. However, MF increases as a function of isocenter to tumor distance (see right panel in Fig. 4), suggesting that farther apart the tumors, the more MUs are required to deliver the target coverage and consequently more low‐dose spillage to the non‐target tissues.

## 4 DISCUSSION

In the present study, we investigated the potential reduction of normal lung dose while utilizing jaw tracking options in the treatment of single‐isocenter/two‐lesion lung SBRT patients. For similar target coverage, our JT‐VMAT plan provided lower dose to lungs, tighter intermediate‐dose spillage and relatively lower dose to OAR compared to no JT‐VMAT plan (see Tables 2, 3, and 4). Most importantly, the low‐dose spillage to the normal lung (V5, V10, V20, MLD, and maximum dose to 1000 cc of lungs) decreased significantly with JT‐VMAT, up to 11% on average, compared to no JT‐VMAT. Similar MF between the two plans suggests that the total number of the MUs remained similar, therefore, the treatment delivery efficiency was not affected by the use of jaw tracking. However, as the distance between the two lesions increased the MF increased and, in general, the low‐dose spillage to the normal lung increased.

A few investigators have reported the dosimetric advantages of jaw tracking techniques for IMRT and VMAT planning.^28^, ^29^, ^30^, ^31^, ^32^ For instance, Joy et al.^32^ has shown the overall reduction of normal tissues V5, V10, and V20 doses by about 2% when applying jaw tracking for the step‐and‐shoot IMRT. Another retrospective study by Kim et al.^31^ assessed the potential advantages of jaw tracking technique by using control point sequence of VMAT planning for head and neck, thoracic, abdominal, and prostate patients. For the head and neck cases, the OAR mean dose was reduced by 4.3% to 12% with jaw tracking. For all prostate patients, the dose reduction was more significant in the dose regions of D80 to D95 compared to D5 to D20 with jaw tracking. Another study by Wu et al.^32^ has shown that maximum and mean doses to the various OAR for head and neck, thoracic, abdominal, and pelvis patients were reduced by up to 7 and 3 Gy, respectively, with artificially locking the jaw coordinates of the jaw tracking VMAT plan. However, in their study, the VMAT plans were not intended for clinical use, but were created for the evaluation of jaw tracking technique on the basis of identical mechanical parameters only. The fixed jaw plans were not optimized using the same objectives for clinical use.

While agreeing with aforementioned retrospective reports, our clinically optimized synchronous JT‐VMAT plan exhibits superior OAR protection for normal lung doses as well as other OAR sparing prospectively compared to no JT‐VMAT for the given complexity of single‐isocenter/two‐lesion lung SBRT setting. By tracking the jaws during SBRT VMAT plan optimization, the magnitude of normal lung dose reductions (the OAR closest to the multiple targets) observed in this study were generally consistent with previous studies,^28^, ^29^, ^30^, ^31^, ^32^, ^33^, ^34^ yet relatively higher differences (up to 11%) were observed, perhaps due to the unique complexity of the clinical situations and the distance between the tumors. It is worthwhile to mention that MLC transmission of our 6 MV‐FFF beam was 1.2% and was modeled by the TPS and incorporated in the dose calculation.

One of the major concerns for treating multiple lung lesions synchronously using single‐isocenter SBRT plan was the non‐trivial low‐dose spillage in the normal lung, such as V20, V10, V5, and MLD, as described above. Per RTOG recommendation, all of our single‐isocenter/two‐lesion JT‐VMAT lung SBRT plans had V20 < 10%–15%. Moreover, for our JT‐VMAT plans normal lung V5 and MLD were kept less than 40% and 6.0 Gy, on average, respectively.^25^, ^26^, ^27^ It was observed that when the isocenter to tumor distance increased, the normal lung V20, V10, V5, MLD, and maximum dose to 1000 cc of normal lung increased. Our treatment planning strategy favored minimizing lung dose with the jaw tracking approach. By selecting patient specific collimator angles in conjunction with jaw tracking the MLC transmission and leakage dose due to the leaves traveling in between two tumors can be minimized. This can potentially help reduce severe lung toxicity with careful attention to normal lung dose parameters such as V5, V10, V20, and MLD during VMAT plan optimization and perhaps JT‐VMAT plan may decrease the probability of developing radiation‐induced acute or late side effects.

In summary, the potential benefit of applying jaw tracking approach in Truebeam (with 6MV‐FFF beam) for single‐isocenter/multitarget lung SBRT setting with curative therapeutic dose of BED > 100 Gy has been presented. It is shown that jaw tracking during SBRT VMAT plan optimization potentially reduces doses to OAR specifically significantly reducing dose to normal lungs while providing similar target coverage. The main advantages of jaw tracking method were more applicable for treating dispersed multiple lesions with relatively higher prescription dose per fraction (longer treatment time) such as the examples presented here or for irregular larger target volume near the critical structures. Therefore, to minimize non‐target dose we strongly recommend jaw tracking approach to be applied during VMAT SBRT plan optimization, thereby reducing the MLC leakage and transmission and potentially minimizing unwanted dose to the patients.

## 5 CONCLUSION

Similar target coverage yet more clinically shaped intermediate dose fall‐off and OAR sparing have been achieved by utilizing the jaw tracking options at Truebeam for 6 MV‐FFF beam during VMAT plan optimization in the treatment of single‐isocenter/two‐lesion lung SBRT. In this setting, the main advantages of jaw tracking options were observed in the low‐dose spillage to the normal lungs. Similar values of MF for JT‐VMAT and no JT‐VMAT suggest that overall treatment time did not increase significantly due to jaw tracking with perhaps similar plan delivery complexity. However, a higher value of MF was observed for the tumors located far from each other, and hence the higher dose to the normal lungs. The reduction of normal lung and OAR dose by jaw tracking during SBRT procedures can potentially reduce the risk of acute/late toxicity.

## CONFLICT OF INTEREST

None.
